# Histone acetyltransferase inhibitor II induces apoptosis in glioma cell lines via the p53 signaling pathway

**DOI:** 10.1186/s13046-014-0108-3

**Published:** 2014-12-19

**Authors:** Li-Xiao Xu, Zhi-Heng Li, Yan-Fang Tao, Rong-Hu Li, Fang Fang, He Zhao, Gang Li, Yan-Hong Li, Jian Wang, Xing Feng, Jian Pan

**Affiliations:** Institute of Pediatric Research, Children’s Hospital affiliated to Soochow University, Suzhou, 215006 China; Department of Hematology and Oncology, Children’s Hospital of Soochow University, Suzhou, 215006 China; Department of Neonatology, Children’s Hospital of Soochow University, Suzhou, 215006 China

**Keywords:** HATi II, Glioma, Apoptosis, LncRNA/mRNA, p53 signaling pathway

## Abstract

**Background:**

Histone acetyltransferase (HAT) inhibitors can inhibit proliferation and induce apoptosis in cancer cell lines. The novel cell-permeable p300/CREB-binding protein (CBP)-selective HAT inhibitor HATi II can reduce histone H3 acetylation and induce chromatin condensation in HeLa cells. Here, we examined the effects and mechanism of action of HATi II in glioma cell lines.

**Methods:**

Cell viability was assessed using the CCK-8 assay. Cell cycle analysis was performed using flow cytometry. Apoptosis was evaluated using Annexin V staining and flow cytometry, Hoechst 33342 staining and the TUNEL assay. Expression and cleavage of caspase-3, caspase-9 and poly ADP-ribose polymerase (PARP) were assessed by Western blotting. Statistical analysis was performed using two-tailed Student’s t-tests. The gene expression profiles of U251 glioma cells treated with HATi II or DMSO were analyzed using the Arraystar Human 8 x 60 K LncRNA/mRNA expression array; data was analyzed using MEV (Multi Experiment View) cluster software. Datasets representing genes with altered expression profiles (≥2-fold) derived from the cluster analyses were subjected to gene ontology and pathway analysis.

**Results:**

HATi II inhibited the proliferation of U251, U87, HS683 and SHG44 cells in a dose-dependent manner. HATi II induced cell cycle arrest at the G2/M phase, and induced significant levels of apoptosis, apoptotic body formation and DNA fragmentation in HATi II-treated U251 and SHG44 cells. HATi II induced cleavage of caspase-3, caspase-9 and PARP in U251 and SHG44 cells. In HATi II-treated U251 cells, 965 genes were upregulated, 984 genes were downregulated and 3492/33327 lncRNAs were differentially expressed. GO analysis showed the differentially expressed genes with known functions are involved in a variety of processes; alcoholism, p53 signaling pathway, cytokine-cytokine receptor interaction and transcriptional mis-regulation in cancer were the four most significant pathways. Upregulation of p53 signaling pathway-related genes in HATi II-treated cells was confirmed by quantitative RT-PCR and Western blotting.

**Conclusions:**

HATi II inhibits proliferation and induces apoptosis via the caspase-dependent pathway in human glioma cell lines, possibly by activating the p53 signaling pathway. HATi II deserves further investigation as a novel treatment for glioma.

**Electronic supplementary material:**

The online version of this article (doi:10.1186/s13046-014-0108-3) contains supplementary material, which is available to authorized users.

## Introduction

Gliomas are the most common type of primary brain tumor in adults and second most common childhood malignancy after leukemia [1,2]. The annual incidence of glioma is approximately 3–8 per 10 million [3]. According to the histopathological and clinical criteria established by the World Health Organization (WHO), gliomas can be grouped into four grades I–IV [4], and there are three principal types of glioma: astrocytomas, oligodendrogliomas and oligoastrocytomas. Despite recent advances in surgical resection, chemotherapy and radiotherapy, the prognosis of patients with glioma continues to be very poor; the five-year overall survival rate for glioma is less than 10% [5] and is the third lowest of all cancers after pancreatic and lung cancer [6-8].

Current standard therapies for newly-diagnosed glioma include surgical resection followed by adjuvant radiotherapy and/or chemotherapy [9]. Nevertheless, surgical resection is often limited by a lack of a clear primary tumor margin and by the location of the tumor close to vital anatomical structures. Moreover, the effectiveness of radiotherapy and chemotherapy is suboptimal in many cases, largely due to the rapid development of tumor cell resistance to radiation and chemotherapeutic drugs. In addition, the blood-brain barrier and side-effects limit the effectiveness of chemotherapy drugs. Clinicians are eager to find novel, more specific therapeutic options with fewer side-effects for glioma; small-molecule inhibitors of molecular targets may be promising candidate drugs.

Recent data has strongly indicated that alterations at the histone level may play a role in glioma tumorigenesis. Histone acetylation is one of the most well-characterized epigenetic modifications and is controlled by histone acetyltransferases (HATs) and histone deacetylases (HDACs) [10]. HATs play a central role in the modification of chromatin to create a favorable environment for a number of important cellular processes [11]. Acetylation neutralizes the positive charge on lysine residues and thereby weakens the bond between DNA and histone tails. Thus, histone acetylation is linked to transcriptional activation, whereas deacetylation is generally associated with transcriptional repression. Deletions or inactivating mutations in multiple genes encoding HATs have been associated with oncogenesis and tumor progression; these mutations alter the transcription of genes that regulate key functions such as proliferation, cell cycle progression and apoptosis [12,13]. Numerous genetic studies have also indicated that HATs are important factors linked to a variety diseases, including cancer [14,15].

Enzymes that catalyze the reversible post-translational modification of histones on lysine residues by acetylation and methylation have received attention as potential drug targets in cancer and other diseases. A recent study found that a variety of small-molecule inhibitors which target HATs could affect cell proliferation and induce tumor cell growth arrest, apoptosis and differentiation in prostate cancer and neuroblastoma cell lines [16-18]. The effects of HATs on cellular physiology and disease could potentially be ameliorated via the use of specific pharmacological inhibitors.

Very few specific HAT inhibitors have been identified; however, several compounds with HAT-inhibitory activity were recently identified. Interestingly, some of these compounds have been shown to inhibit the growth of cancer cells [19]. Anacardic acid, isolated from cashew nut shell liquid, was identified as a potent non-competitive inhibitor of both p300 and p300/CBP-associated factor (PCAF) HAT activity *in vitro* [20]. Quinoline was reported to promote tumor cell apoptosis in human leukemia cell lines by inhibiting p300 HAT activity [21]. Another p300/CBP HAT inhibitor compound, C646, could inhibit the growth of both human melanoma and non-small-cell-lung (NSCL) cancer cell lines [22], and also could inhibit the growth of primary blasts isolated from patients with t(8;21)-positive acute myelocytic leukemia (AML) as well as Kasumi-1 cells [23].

Histone acetyltransferase inhibitor II (HATi II) is a novel cell-permeable bis-arylidene cyclohexanone compound that acts as a p300/CBP-selective HAT inhibitor, which can reduce histone H3 acetylation and induce chromatin condensation in HeLa cells. The p300 protein is a transcriptional co-activator with intrinsic HAT activity that plays a crucial role in cell cycle progression, differentiation and apoptosis. Inhibition of p300 suppresses the cellular growth of melanoma cells [24] and induces apoptosis in prostate cancer cells [25]. P300 activity is also required for the G1/S transition in cancer cells [26,27]. Despite the fact that the anti-tumor effects of p300 inhibitors have been reported in other cancers, the effect of inhibiting p300 has not been extensively investigated in glioma cells. In the present study, we examined the molecular function of HATi II in glioma cell lines, and observed that HATi II can inhibit proliferation and induce cellular apoptosis via the caspase-dependent apoptotic pathway. In addition, microarray analysis and quantitative real-time PCR indicated that HATi II activates the p53 signaling pathway in glioma cells. These results suggest that HATi II may represent a novel target for therapy for patients with glioma.

## Materials and methods

### Reagents

HATi II was purchased from Calbiochem (Billerica, MA, USA) and dissolved in DMSO (Sigma-Aldrich, St. Louis, MO, USA). The Cell Counting Kit-8 was obtained from Dojindo Laboratories (Kumamoto, Japan); 4,6-diamidino-2-phenylindole (DAPI) and Hoechst 33342 were purchased from Sigma-Aldrich. Mouse monoclonal antibody against β-actin and rabbit polyclonal antibodies against caspase-3, caspase-9, PARP, PTEN and CDK1 were purchased from Cell Signaling Technology (Beverly, MA, USA); Reprimo, RRM2, CCNE2 and SFN from Boster (Wuhan, China); and p53 and p21 from Beyotime (Jiangsu, China). Anti-mouse and anti-rabbit peroxidase conjugated secondary antibodies were purchased from Pierce (Madison, WI, USA).

### Cell culture

The glioma cell lines U251, U87, HS683 and SHG44 were obtained from the Cell Bank of the Chinese Academy of Sciences (Shanghai, China). The cells were maintained in DMEM (Invitrogen Life Technologies, Paisley, UK) containing 10% fetal bovine serum (FBS; Hyclone, UT, USA) at 37°C in 5% CO_2_. The cells were confirmed to be free from mycoplasma every three months using a commercially available kit (Invitrogen, Shanghai, China). All of the researchers who contributed to this study received standard cell culture training to prevent cell cross-contamination. The study was approved by the Ethics Committee of Children's Hospital of Soochow University.

### Cell proliferation and viability assays

Glioma cells (2 × 10^4^) were seeded in 96-well plates, cultured overnight, and then incubated with DMSO or varying concentrations of HATi II (2.5, 5, 7.5, 10, 12.5, 15, 17.5, 20 or 25 μM) for 48 h; the same volume of DMSO was added to the vehicle-treated wells as the drug-treated wells, and each drug concentration was tested in three replicate wells. Then, 10 μL CCK8 solution (DOJINDO, Shanghai) was added to each well, incubated at 37°C for 4 h and the optical density (OD) values were measured at 450 nm using a scanning multi-well spectrophotometer. The survival rate of the treated cells was calculated relative to the control group. Cell proliferation was calculated as a percentage of the DMSO-treated control wells; IC_50_ values were derived after plotting the proliferation values on a logarithmic curve.

### Western blot analysis

Glioma cells were harvested, washed and lysed using 1x RIPA buffer. Protein concentrations were determined using the BCA protein assay kit. Equal amounts of protein from the cell lysates were separated on 10% SDS polyacrylamide gels and transferred onto polyvinylidene fluoride membranes. Membranes were blocked with 5% non-fat milk in TBST and incubated with primary antibodies against caspase 3, caspase 9, PARP, PTEN, CDK1, Reprimo, RRM2, CCNE2 and SFN at 4°C overnight. Then, the membranes were washed and incubated with secondary antibodies against rabbit or mouse IgG conjugated to horseradish peroxidase (Cell Signaling) for 1 h, then washed and the bands were visualized using chemiluminescence (ECL, Amersham-Pharmacia, Uppsala, Sweden); β-actin was used as an internal loading control. The expression of the target proteins relative to β-actin was used as a semi-quantitative comparison between different groups.

### Cell cycle assays

Cells were seeded in 6-well plates (1 × 10^6^ cells/well). HATi II stock solution (10 mM in anhydrous DMSO) was directly added to the culture media to achieve the desired concentrations; the volume of DMSO was kept constant at 0.1%. After treatment with HATi II or DMSO for 24 or 48 h, the cells were harvested and subjected to the following assays. For the cell cycle assay, the cells were washed twice with ice cold PBS, fixed in 70% ethanol at 4°C overnight, incubated with 10 mg/mL RNase A (Sigma-Aldrich) at 37°C for 30 min, and then incubated with 50 mg/mL propidium iodide (Sigma-Aldrich). Cell cycle distribution was assessed by flow cytometry (FC500, Beckman Coulter, FL, USA).

### Cell apoptosis assays

Apoptosis assays were conducted using the BD Annexin V Staining Kit (BD Biosciences, Franklin Lakes, USA) according to the manufacturer’s instructions. Briefly, the cells were washed twice with cold PBS, resuspended in 1× Binding Buffer at a concentration of ~1 × 10^6^ cells/ml, 100 μl of the cell suspension (~1 × 10^5^ cells) was transferred to a 5 ml culture tube, 5 μl Annexin V and PI were added, the cells were gently mixed, incubated for 15 min in the dark at RT, 400 μl of 1× Binding Buffer was added to each tube, and the cells were immediately analyzed by flow cytometry (within 1 h).

### Hoechst 33342 staining analysis

Cells were seeded into 6-well plates, and then treated with HATi II and cultured at 37°C for different periods of time, stained with 0.1 μg/ml Hoechst 33342 (Sigma) for 5 min, then observed by fluorescence microscopy using appropriate filters for blue fluorescence.

### TUNEL assay

DNA double-strand breaks occur late in the apoptotic pathway and were assessed using the TUNEL Apoptosis Detection Kit (Cat: KGA704; Kengent, Nanjing, China). Firstly, the cells were seeded on coverslips, treated with 10 μmol/L HATi II for 48 h, washed, fixed and stained as per the manufacturer’s instructions, and the cell nuclei were stained with 1 μg/mL 4’,6-diamidino-2-phenylindole (DAPI). Apoptotic cells were observed by laser scanning confocal microscopy (OLYMPUS IX71) at an excitation wavelength of 515-565 nm.

### RNA extraction, RNA quality control and microarray hybridization

Total RNA was extracted from the cells using the single-step TRIzol RNA extraction kit (Invitrogen, CA, USA) according to the manufacturer’s instructions, concentrated by isopropanol precipitation and column-purified using the Qiagen RNeasy Mini Kit (Cat No. 74104). The integrity and concentration of the RNA samples were assessed prior to labeling; RNA was quantified and assessed using a NanoDrop ND-1000 (Thermo Scientific), RNA integrity was assessed using denaturing agarose gel electrophoresis. The Quick Amp Labeling Kit (Agilent Technologies) was used for sample labeling and hybridization to Arraystar_Human_LncRNA_8 × 60 k v3.01 microarrays was performed in Agilent Sure Hyb Hybridization Chambers.

### Data collection, normalization and analysis

After washing, the hybridized microarrays were scanned using an Agilent DNA Microarray Scanner and data was extracted using Agilent Feature Extraction software. Quantile normalization and subsequent data processing were performed using the GeneSpring GX v12.0 software package (Agilent Technologies). After quantile normalization of the raw data, mRNAs that had ‘Present’ or ‘Marginal’ flags in at least one out of two samples were selected for differentially expressed mRNA screening. To identify differentially expressed mRNAs, we performed fold change filtering between the two samples using a threshold fold change of ≥ 2.0. Further data analysis was performed using Agilent GeneSpring GX v12.0 software; this analysis was performed by KangChen Biotech, Shanghai, PR China.

### Gene ontology analysis and pathway analysis

Gene ontology (GO) analysis is a functional analysis that associates differentially expressed mRNAs with GO categories. The GO categories were derived from Gene Ontology (www.geneontology.org), which comprises three structured networks of defined terms that describe gene product attributes. The ontology covers three domains: biological process, cellular component and molecular function. Fisher’s exact test was used to determine whether the overlap between the differentially expressed (DE) list and the GO annotation list was greater than that expected by chance. The *P*-value denotes the significance of the GO term enrichment in the DE genes after accounting for the false discovery rate. The lower the *P*-value, the more significant the GO term (a *P*-value ≤ 0.05 is recommended).

Pathway analysis is a functional analysis that maps genes to KEGG (Kyoto Encyclopedia of Genes and Genomes) pathways (http://www.genome.jp/kegg/). The *P*-value (EASE-score, Fisher’s exact test *P* value, or hypergeometric *P*-value) denotes the significance of the pathway correlated to the conditions. The lower the *P*-value, the more significant the correlation (the recommended *P*-value cut-off is 0.05).

### Real-time PCR verification of differentially expressed target genes

Total cellular RNA was isolated using TRIzol reagent (Invitrogen, CA, USA ) according to the manufacturer’s protocol and 1 μg of total RNA was reverse transcribed to cDNA using the ReverTra Ace qPCR RT Kit (Toyobo, Osaka, Japan) following the manufacturer’s instructions. Real-time PCR array analysis was performed in a total volume of 20 μl, including 2 μl of cDNA, primers (0.2 mM each) and 10 μl of SYBR Green mix (Roche). Reactions were run on an Light cycler 480 (Roche) using universal thermal cycling parameters (95°C for 5 min, 45 cycles of 10 sec at 95°C, 20 sec at 60°C and 15 sec at 72°C; melting curve: 10 sec at 95°C, 60 sec at 60°C and then continued melting). Results were obtained using the sequence detection software of the Light cycler 480 and analyzed using Microsoft Excel. The sequences of the primers for the seven target genes and the internal control gene (*GAPDH*) are listed in Additional file 1: Table S1.

### Statistical analysis

The mean ± SD values presented in the figures were calculated from three or more independent experiments. Comparisons between groups were evaluated using two-tailed Student’s *t*-tests; *p* < 0.05 was considered statistically significant.

## Results

### HATi II inhibits the proliferation of glioma cells

**Figure 1:**
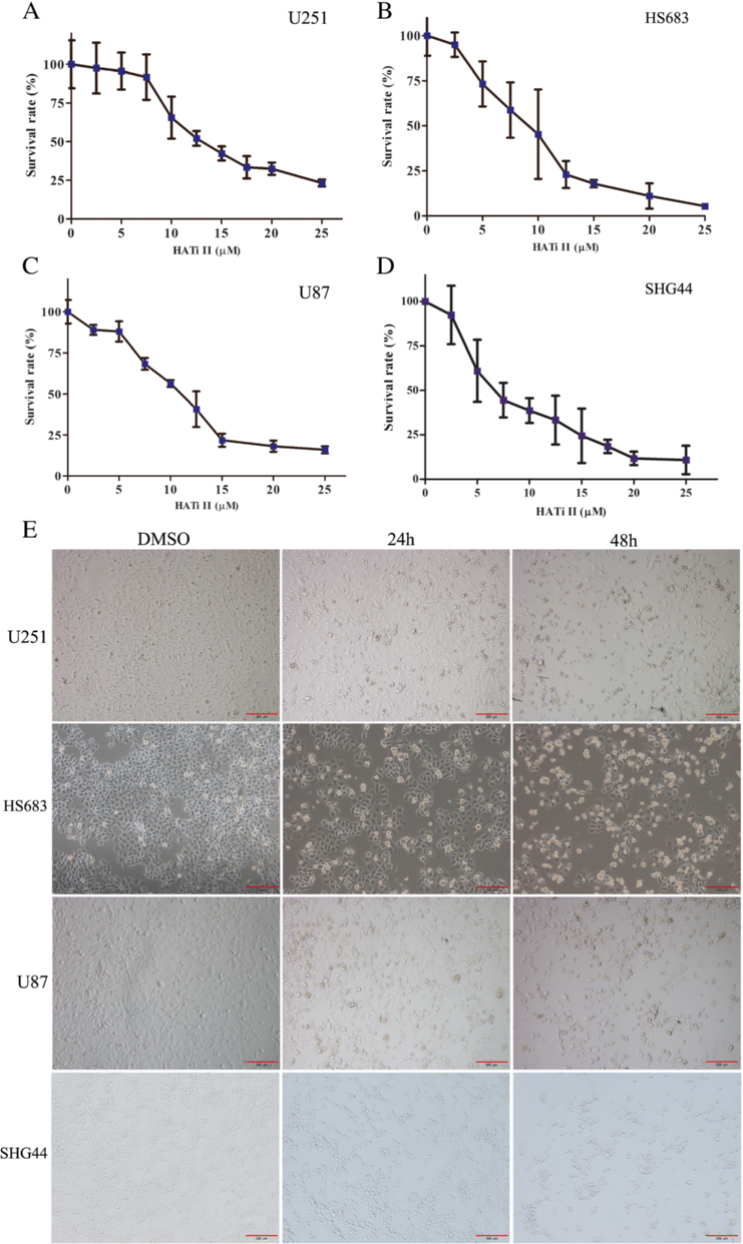
**Growth inhibitory effects of HATi II in glioma cell lines. (A-D)** Cell viability assay. Glioma cells cultured in 96-well plates were treated with different concentrations of HATi II or DMSO for 48 h, and cell viability was determined using the CCK-8 assay. Cell proliferation was calculated as a percentage of the DMSO-treated control cells; IC50 values were derived by plotting the proliferation values on a logarithmic curve. The experiments were repeated three times. **(E)** Phase-contrast microscopy of U251, HS683, U87 and SHG44 cells treated with HATi II for 24 or 48 h (×100).

U251, U87, SHG44 and HS683 cells were treated with different concentrations of HATi II (0, 2.5, 5, 7.5, 10, 12.5, 15, 20 or 25 μmol/L) for 48 h, then cell proliferation was assessed using the CCK-8 kit. HATi II inhibited proliferation in a dose-dependent manner in all four glioma cell lines; DMSO (≤0.1%) had negligible influence on cell proliferation. The IC_50_ values for HATi II at 48 h in U251, U87 and HS683 cells were approximately 12.17 ± 0.7489 μmol/L, 9.513 ± 0.8632 μmol/L and 9.558 ± 1.081 μmol/L; SHG44 cells were more sensitive to HATi II than the other three cell lines with an IC_50_ of 5.9688 ± 0.5351 μmol/L (Figure 1A-1D).

Under the phase-contrast microscope, we observed that treatment with HATi II markedly decreased the numbers of U251, HS683, U87 and SHG44 cells, and a large proportion of the remaining cells became rounded and smaller in size indicating that the cells were undergoing apoptosis (Figure 1E).

### HATi II induces apoptosis in U251 and SHG44 cells

**Figure 2:**
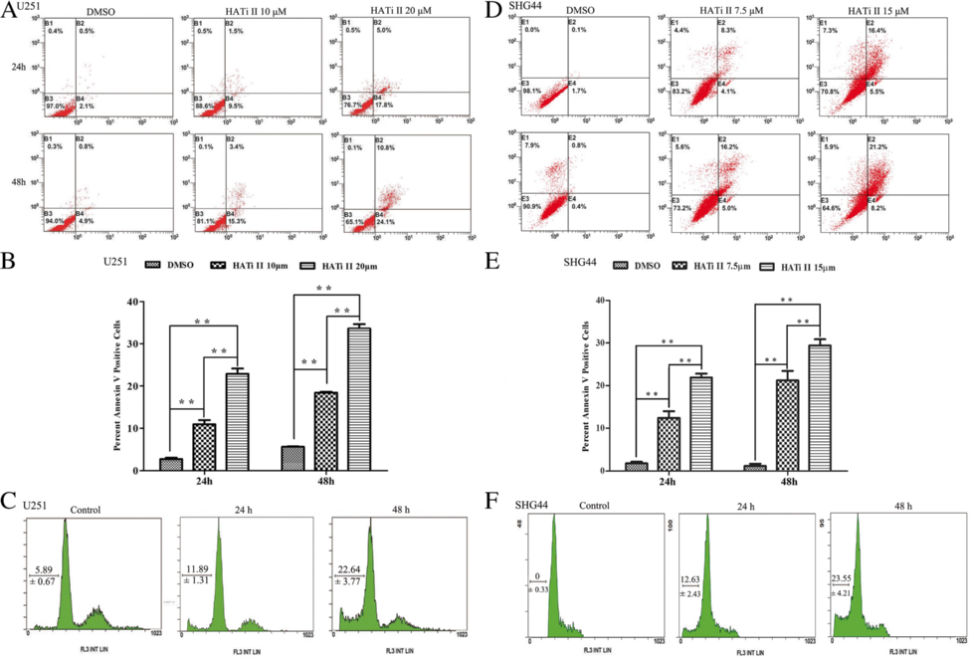
**HATi II induces apoptosis in glioma cell lines. (A-C)** U251 cells or **(D-F)** SHG44 cells were treated with HATi II for 24 or 48 h and apoptosis was determined by Annexin V-FITC/PI dual labeling and flow cytometry **(A, D)**, and the numbers of Annexin V (+) and PI (-)% cells were quantified **(B, E)**; *p < 0.05 or **p < 0.01 compared with DMSO-treated control cells. **(C, F)** Cell cycle analysis of U251 and SHG44 cells treated with HATi II for 24 or 48 h; DNA fragmentation was observed after 24 h and increased in a time-dependent manner. These analyses were repeated three times.

To confirm whether HATi II induces apoptosis in glioma cells, we subjected HATi II-treated U251 cells to the Annexin V assay. Flow cytometry demonstrated that treatment with 10 or 20 μM HATi II for 24 or 48 h significantly increased the numbers of apoptotic cells compared to control cells. The percentage of apoptotic cells in the HATi II 10 μM group was 11% ± 2.2% at 24 h and 22.8% ± 1.7% at 48 h, the percentage of apoptotic cells in the HATi II 20 μM group was 18.7% ± 0.8% at 24 h and 34.9% ± 3.3% at 48 h, compared to 3.6% ± 1.02% at 24 h and 5.7% ± 0.56% at 48 h for control cells (*P* < 0.01; Figure 2A and 2B). Cell cycle alterations were confirmed using the cell cycle assay (Figure 2C). As expected, DNA fragmentation was observed and increased in a time dependent manner. The number of apoptotic cells in the HATi II 10 μM group was 11.89% ± 1.7% at 24 h and 22.64% ± 4.4% at 48 hours; similar results were obtained for SHG44 cells treated with 7.5 or 15 μM HATi II for 24 or 48 h (Figure 2D–F).

**Figure 3:**
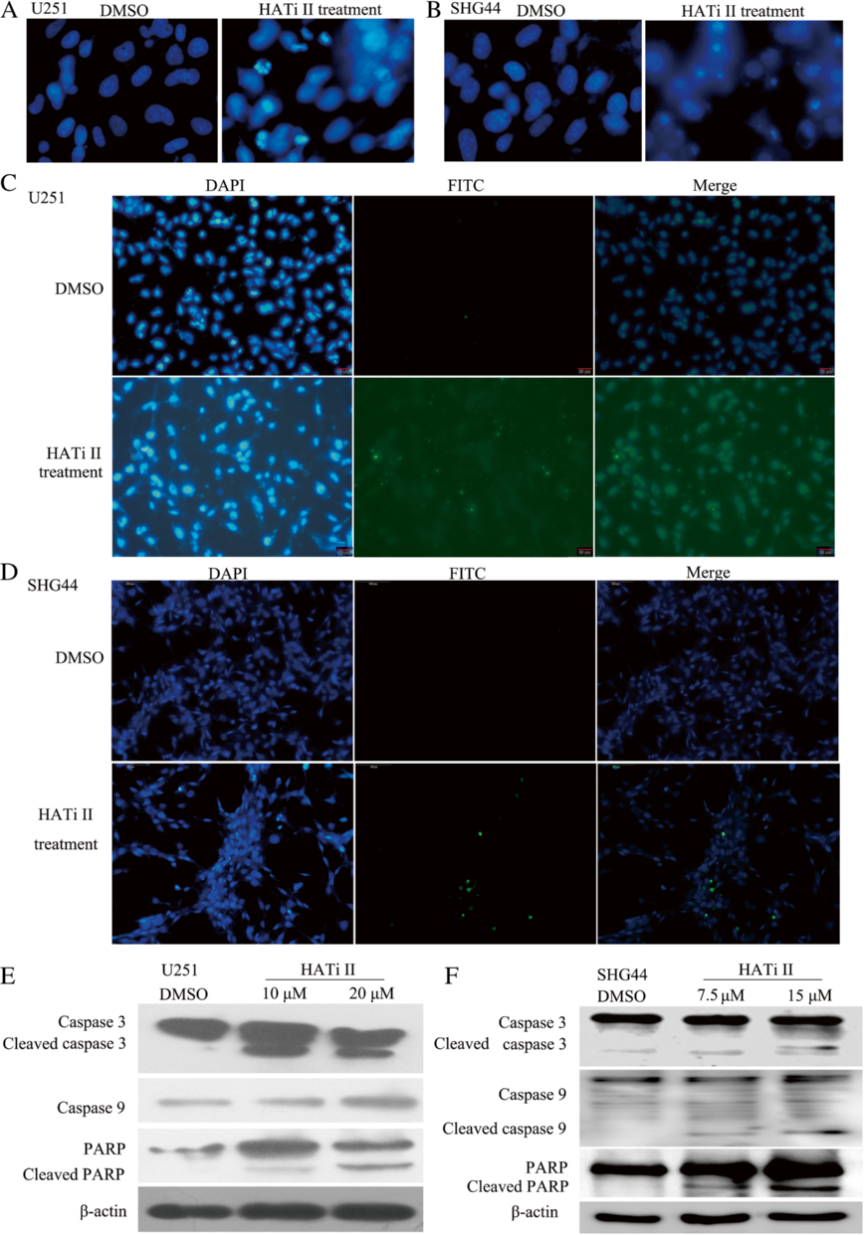
**HATi II induces apoptosis**, **DNA fragmentation and activation of caspase**-**3 in glioma cell lines. (A, B)** The morphologic changes in U251 **(A)** and SHG44 cells **(B)** treated with HATi II were evaluated using Hoechst 33342 staining and fluorescence microscopy. **(C, D)** Apoptosis was assessed by terminal deoxynucleotidyl transferase mediated dUTP nick end-labeling (TUNEL) analysis in U251 **(C)** and SHG44 cells **(D)** treated with HATi II. Apoptotic cells (green) were detected by laser scanning confocal microscopy at an excitation of 515-565 nm, while the cell nuclei were stained with DAPI. The two images have been superimposed to show the apoptotic cells (green) and their position. **(E and F)** Western-blot analysis of the activation of caspase-3, caspase-9 and PARP in U251 **(E)** and SHG44 cells **(F)** treated with HATi II for 48 h.

As shown in Figure 3A and 3B, apoptotic bodies were clearly observed in U251 and SHG44 cells that had been treated with HATi II for 48 h and then stained with Hochest 33342. Additionally, the TUNEL assay showed that HATi II markedly increased the number of apoptotic cells relative to the respective control U251 and SHG44 cells treated with DMSO (Figure 3C and 3D). These results were consistent with the Annexin V assay and cell cycle analysis, and confirmed that HATi II induces apoptosis in U251 and SHG44 cells. We obtained similar results in U87 and HS683 treated with HATi II (Additional file 2: Figure S1).

### HATi II induces caspase-dependent apoptosis in glioma cells

As we observed that HATi II induced apoptosis in glioma cells, we examined the cleavage of PARP, caspase-9 and caspase-3 by Western blotting. The expression of cleaved caspase-3, caspase-9 and PARP increased markedly in U251 cells treated with 10 or 20 μM HATi II for 48 h and SHG44 cells treated with 7.5 or 15 μM HATi II for 48 h (Figure 3E and 3F). Additionally, caspase-3 and PARP underwent cleavage in a dose-dependent manner in U251 and SHG44 cells treated with HATi II. These results confirmed that HATi II induces apoptosis in glioma cells by activating the caspase-dependent pathway. We obtained similar results in U87 and HS683 cells treated with HATi II (Additional file 3: Figure S2).

### Microarray analysis of differentially expressed genes in HATi II-treated U251 cells

**Figure 4:**
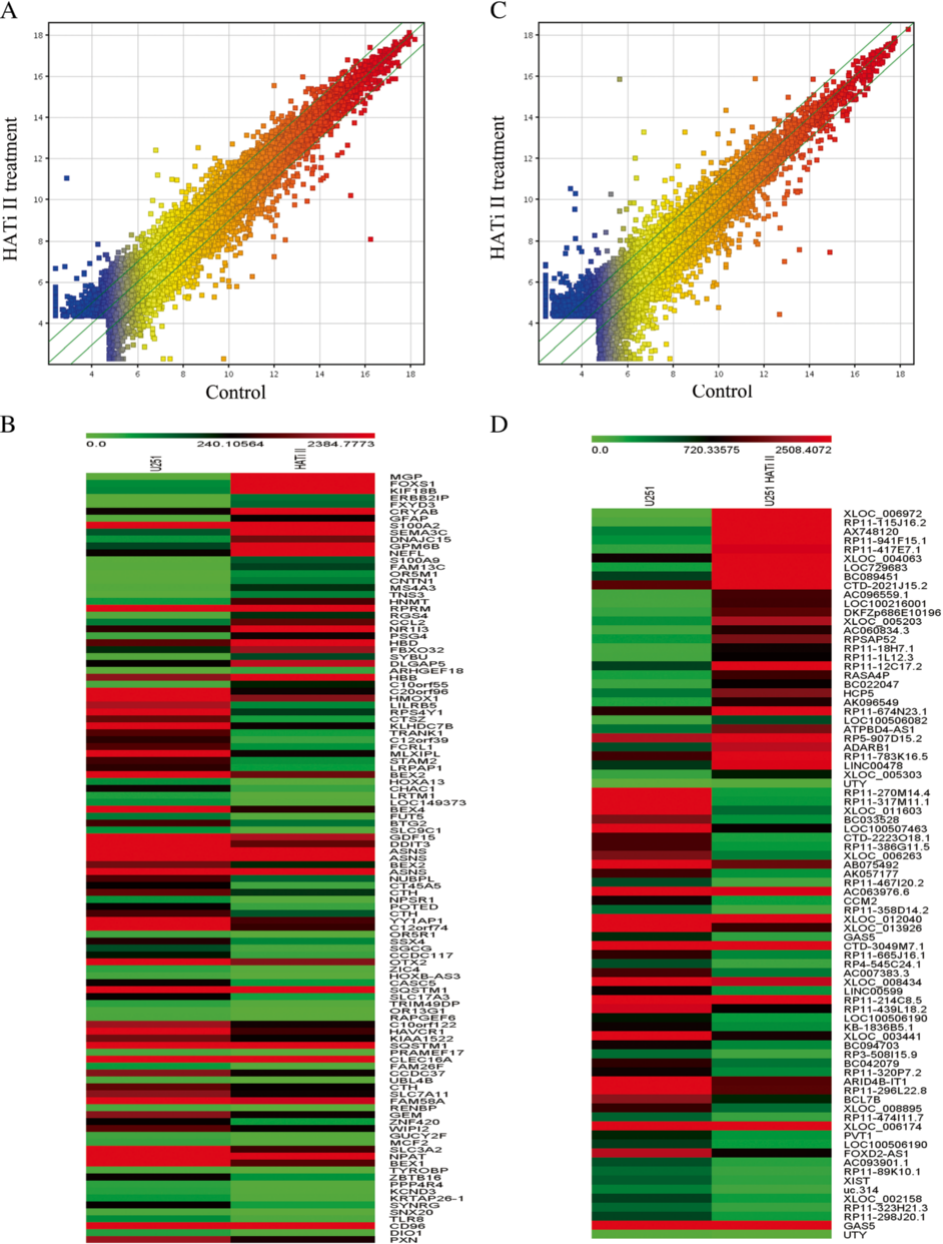
**Microarray analysis of the expression profiles of HATi II**-**treated and DMSO-** **treated U251 cells. (A, B)** The box plot is a convenient way to quickly visualize the distributions of a dataset for mRNA **(A)** and lncRNA **(B)** profiles. The distributions of the log_2_ ratios between the groups after normalization are presented. **(C)** Differentially expressed mRNAs and **(D)** differentially expressed lncRNAs were analyzed using hierarchical clustering. Hierarchical clustering analysis arranges samples into groups based on their expression level, which allows us to evaluate the relationships between samples. “Red” indicates high relative expression, and “green” indicates low relative expression (mRNA fold changes ≥ 5 and lncRNA fold changes ≥ 3).

The Arraystar_Human_LncRNA_8x60k v3.0 1 microarray was used to identify differentially expressed lncRNA/mRNA in HATi II-treated U251 cells for 48 h compared to DMSO-treated control cells. The threshold of volcano plot filtering used to screen the differentially expressed mRNAs was a fold change > 2.0. In the lncRNA/mRNA expression profiling data, we identified a total of 23773 mRNAs and 1949 differently expressed mRNAs in HATi II-treated U251 cells (Figure 4A, Additional file 4: Table S2 and Additional file 5: Table S3). Compared to DMSO-treated control cells, 965 mRNAs were significantly upregulated and 984 mRNAs were significantly downregulated in HATi II-treated U251 cells. Clustering analysis was used to visualize the relationships between the mRNA expression patterns present in the samples (fold changes ≥ 5; Figure 4B).

In the lncRNA expression profiling data, we found a total of 33327 long non-coding RNAs (lncRNAs) expressed in U251 cells, of which 3492 were differently expressed in HATi II-treated cells (Figure 4C, Additional file 6: Table S4 and Additional file 7: Table S5). Hierarchical clustering analysis of the differently expressed lncRNAs with a fold change ≥ 3-fold is presented in Figure 4D. A total of 743 lncRNAs close to coding genes were identified to be changed after HATi II treatment (Additional file 8: Table S6).

### Gene ontology analysis of differentially expressed mRNA s

We performed ontologic pathway enrichment analysis for the differently expressed genes and gene product enrichment with particular attention to GO biological processes and molecular function. Fisher’s exact test was used to determine whether the overlap between the differentially expressed gene list and the GO annotation list was greater than that expected by chance (a *P*-value ≤ 0.05 is recommended).

**Figure 5:**
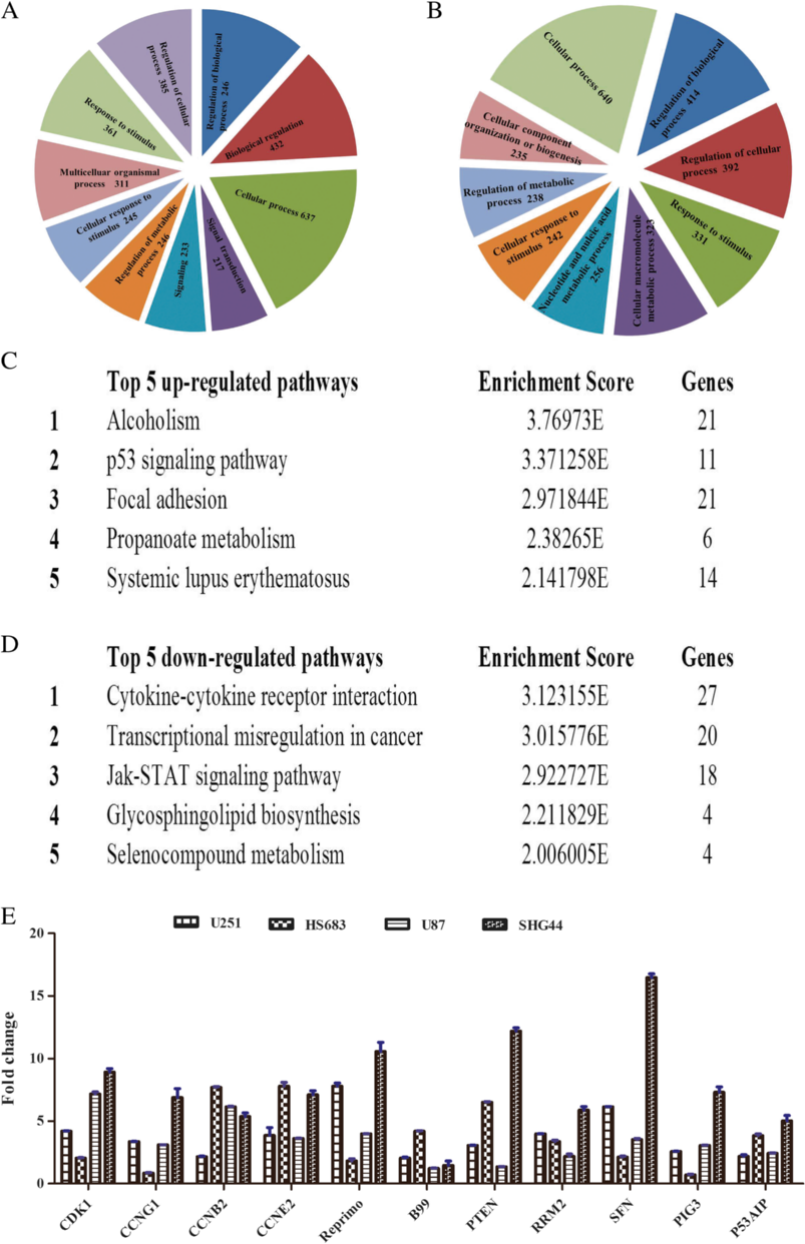
**Bioinformatic analysis of the differentially expressed genes in HATi II-** **treated U251 cells.** Gene ontology enrichment analysis provides a controlled vocabulary to describe the attributes of differentially expressed transcripts in all organisms (*p*-values ≤ 0.05 are recommended). **(A)** Upregulated genes and **(B)** downregulated genes in HATi II-treated U251 cells. Pathway analysis is a functional analysis that maps genes to KEGG pathways. The top five upregulated pathways **(C)** and top five downregulated pathways **(D)** in HATi II-treated U251 cells are listed. The “p53 signaling pathway”, which is associated with apoptosis, was activated in HATi II-treated U251 cells. **(E)** Confirmation of the microarray results. The expression of p53 signaling pathway-related genes in HATi II-treated U251, HS683, U87 and SHG44 cells was validated by quantitative real-time PCR. The columns represent the log-transformed median fold changes in expression relative to DMSO-treated cells.

We found that the most enriched GOs targeted by the upregulated and downregulated transcripts were involved in a variety of functions including cellular processes, biological regulation, cell cycle, response to stimulus, immune and defense response, signal transduction, transcriptional regulation and metabolism (Figure 5A and 5B). The genes associated with the most enriched GO terms in HATi II-treated U251 cells were linked to DNA ligation, regulation of protein processing, cell cycle and mitosis, and oxygen transporter activity, and included *HBMG2*, *CRCC4*, *TOP2A*, *RAD51*, *A2M*, *C3*, *F12*, *KLKB1*, *GTSE1*, *CETN2*, *PLK4*, *KLF11*, *KLF3B* and *HBM* (Additional file 9: Table S7). In contrast, the genes associated with the most enriched GO terms in DMSO-treated U251 control cells included factors involved in response to stimulus, immune system and defense processes, and included *S100A7*, *MMP3*, *BCL2*, *HMOX1*, *APCS*, *IGF1*, *SP3*, *FOS*, *CXCL1*, *TIMP3*, *DUSP6*, *MAPK10*, *CLEC7A* and *TLR8* (Additional file 10: Table S8).

### Pathway analysis and certification

The KEGG database was used to investigate the pathways in which the differentially expressed genes are involved. KEGG pathway annotations of the five most enriched pathways are shown in Figure 5C and 5D. Specifically, the upregulated pathways included alcoholism, p53 signaling pathway, focal adhesion, propanoate metabolism, cell cycling, regulation of actin cytoskeleton and ECM-receptor interactions. One of these pathways, the p53 signaling pathway, contained 11 genes that were upregulated in HATi II-treated cells.

To validate the results of the mRNA microarray assay, quantitative RT-PCR analysis was performed to confirm the expression of genes related to the p53 signaling pathway in HATi II-treated U251, HS683, U87 and SHG44 cells and DMSO-treated control cells. Quantitative RT-PCR demonstrated that *CDK1*, *CCNG1*, *CCNE2*, *CCNB2*, *Reprimo* (*RPRM*), *GTSE1* (*B99*), *PTEN*, *SFN* (*stratifin*), *RRM2*, *TP53I3* (*PIG3*) and *P53AIP* were upregulated by HATi II (Figure 5E); these results were mostly consistent with the results of microarray analysis.

**Figure 6:**
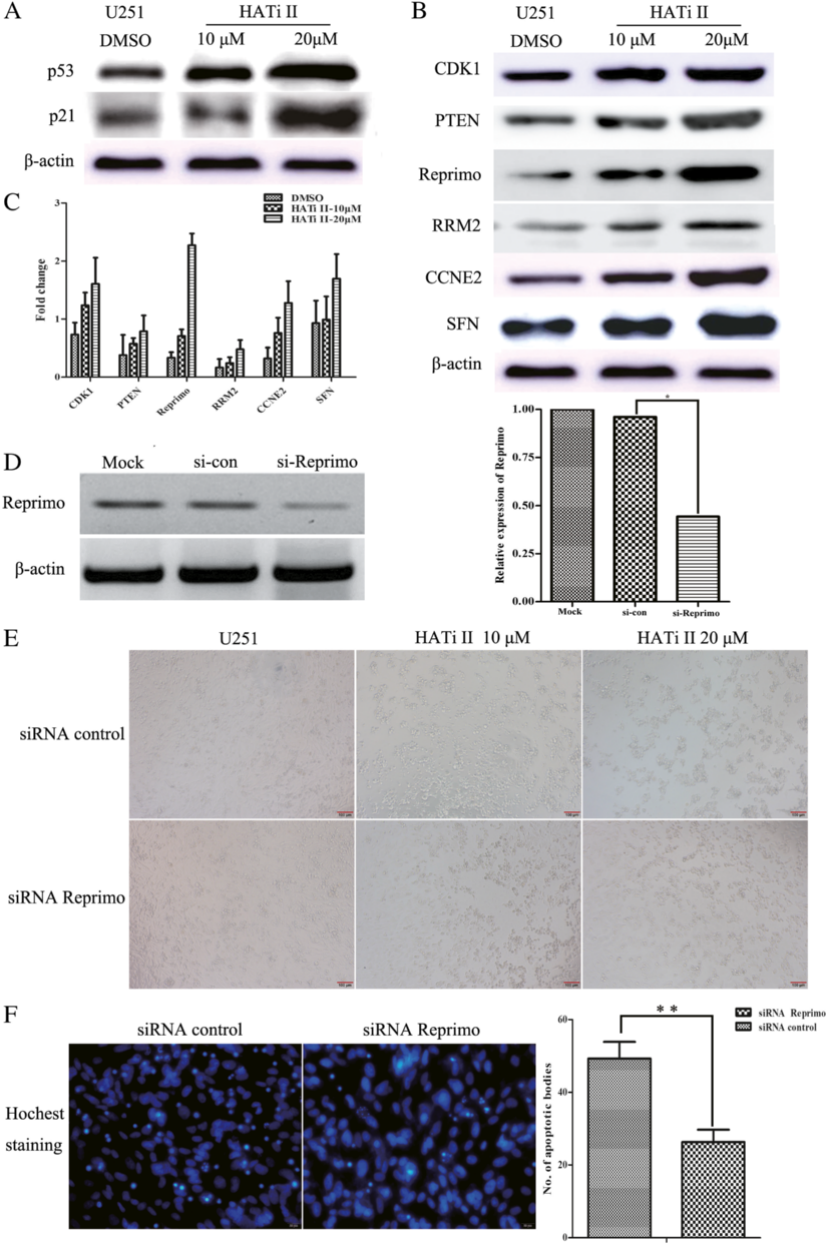
**Protein expression of factors related to the p53 signaling pathway in U251 cells treated with HATi II.** Western blot analysis of p53 and p21 **(A)** and factors downstream of the p53 signaling pathway **(B, C)** in U251 cells treated with HATi II. **(D)** Western blot analysis confirming siRNA-mediated knockdown of Reprimo. **(E)** The morphological assay indicated that knockdown of Reprimo markedly reduced cell death in a dose-dependent manner in U251 cells treated with 10 or 20 μmol/l HATi II for 48 h. **(F)** The hoechst 33342 staining assay indicated that knockdown of Reprimo reduced apoptosis in U259 cells treated with 10 μmol/l HATi II for 48 h compared with control cells; **P < 0.01.

Significant changes in the protein levels of factors related to the p53 pathway were also observed in U251 cells treated with HATi II, as shown in Figure 6A and 6B. P53 and p21 were obviously upregulated by HATi II treatment in a dose-dependent manner. A number of proteins downstream of the p53 signaling pathway, such as CDK1, CCNE2, Reprimo, PTEN, SFN and RRM2, were also significantly increased in a dose-dependent manner in U251 cells treated with HATi II, compared to control cells (Figure 6B and 6C). These results indicate that HATi II may activate p53 and promote transcription and activation of genes downstream of the p53 pathway. As shown in Figure 6A and 6B, Reprimo was significantly upregulated by HATi II at both the mRNA and protein level. To determine the function of Reprimo in glioma cells treated with HATi II, we knocked down the expression of Reprimo in U251 cells using a siRNA. Silencing of reprimo attenuated HATi II-induced cell death, (Figure 6E) and reduced the number of apoptotic cells by almost 25% (Figure 6F).

## Discussion

At present, the therapeutic options for glioma are insufficient, and more effective therapeutic options are urgently required; small-molecule inhibitors may represent specific, less toxic candidate drugs. Recent research has indicated that a variety of small-molecule inhibitors that target HATs can induce growth arrest, inhibit proliferation, induce apoptosis or affect differentiation in tumor cells [16-18]. HATi II, a novel p300/CBP-selective HAT inhibitor reduced histone H3 acetylation and induced chromatin condensation in HeLa cells. In this study, we investigated the effects and mechanism of action of HATi II in glioma cell lines.

In this study, we demonstrated that HATi II can inhibit the growth of glioma cell lines, as well as neuroblastoma and Wilms tumor cell lines (data not shown), which suggests that HATi II exerts broad spectrum anti-cancer activity. P300 is required for orderly G1/S cell cycle phase transition in human cancer cells, and inhibition of p300 blocks progression into the S-phase of cell cycle [26,28]. The p300/CBP HAT inhibitor compound C646 has been shown to inhibit the growth of both melanoma and non-small-cell-lung (NSCL) cancer cell lines [22]. In our study, the CKK-8 assay, Hoechst 33342 staining, the Annexin V assay, flow cytometry and the TUNEL assay demonstrated that HATi II inhibited proliferation and induced cell cycle arrest and apoptosis in glioma cell lines. DNA fragmentation was observed after 24 h in HATi II-treated cells, and increased in a time-dependent manner. In prostate cancer cells, inhibition of p300 induced apoptosis via multiple pathways, and also decreased the expression of MMP-2 and MMP-9, which reduced the migratory and invasive ability of the cells [25].

Apoptosis is a genetically controlled mechanism of cell death that is essential for the elimination of unwanted cells during normal development and for the maintenance of tissue homeostasis [29,30]. Classical caspase-dependent apoptosis requires proteolytic activation of caspases that are synthesized as latent proenzymes. Once activated, caspases cleave a wide range of molecules, eventually resulting in cellular destruction [31]. Cleavage of PARP by caspases is considered to be a hallmark of apoptosis. In our experiments, HATi II increased the expression of cleaved PARP, caspase-9 and caspase-3 in U251 and SHG44 cells. Cleavage of caspase-3 and PARP is indicative of apoptosis and confirmed that HATi II induced apoptosis via the caspase-dependent pathway. This observation is in keeping with recent reports of the proapoptotic activity of NU9056, which is a histone acetyltransferase inhibitor. NU9056 inhibits cell growth and induces apoptosis via caspase activation in prostate cancer cell lines [32]. In addition, it is noteworthy that both cell cycle arrest and apoptosis were observed in neuroblastoma cell lines (IMR-32 and SH-SY5Y) treated with HATi II (data not shown), which suggests that HATi II has potential for use in the clinic.

Microarray analysis demonstrated that 965 mRNAs were significantly upregulated and 984 mRNAs were significantly downregulated (≥2.0-fold) in HATi II-treated U251 cells. Additionally, 3492 lncRNAs were identified to be differently expressed in HATi II-treated U251 cells. In recent years, multiple studies have demonstrated that nearly every step in the life cycle of a gene can be regulated by lncRNAs [33]. Nevertheless, the role of lncRNAs in apoptosis not well-studied. In this study, a total of 743 lncRNAs close to coding genes were identified to changed in HATi II-treated cells. Serine/threonine kinase 40 (*STK40*), a negative regulator of NF-kappa-B and p53-mediated gene transcription [34], is the coding gene closest to lncRNA chr1:36804800-36856000+, which was upregulated 10-fold after HATi II treatment. BCL2-associated athanogene 6 (*BAG6*), the coding gene closest to lncRNA NR_002812 that was upregulated 5–fold by HATi II, is implicated in the regulation of apoptosis. After DNA damage, BAG6 accumulates in the nucleus and forms a complex with p300/EP300, enhancing p300/EP300-mediated p53/TP53 acetylation leading to increased p53/TP53 transcriptional activity [35,36]. In addition, *RASSF1* (Ras Association (RalGDS/AF-6) Domain Family Member 1) is the coding gene closest to the downregulated lncRNA BC033528, RASSF1 has been shown to induce cell cycle arrest and is required for death receptor-dependent apoptosis [37]. Therefore, these results indicate that lncRNAs may play a role in HATi II-induced apoptosis in glioma cell lines.

GO analysis showed that the differentially expressed mRNAs with known functions are involved in a variety of processes including cellular processes, biological regulation, cell cycle, response to stimulus, immune and defense response, signal transduction, transcriptional regulation and metabolism. Numerous signaling pathways and molecules are involved in apoptosis [29,38]. One major apoptotic signaling pathway involves the p53 tumor suppressor. KEGG pathway annotation demonstrated that the p53 signaling pathway was activated by HATi II, which suggested that HATi II may induce apoptosis in U251 cells via activation of the p53 signaling pathway. Based on our analysis of this analysis, quantitative real-time PCR was used to verify the expression of a number of crucial target genes downstream of p53, to test the hypothesis that HATi II induces p53 transcription-dependent death and exclude the possibility of false positive results from the microarray.

A number of differently expressed genes were chosen as markers of the transcriptional function of p53. *CDK1*, *CCNG1*, *CCNE2*, *CCNB2* and *p53* are transcriptionally regulated at different phases of the cell cycle. Reprimo appears to induce cell cycle arrest by inhibiting CDK1 activity and nuclear translocation of the CDC2 cyclin B1 complex, and may be involved in regulation of p53-dependent G2 cell cycle arrest. *B99* is only expressed in the S and G2 phases of the cell cycle, and in response to DNA damage, it accumulates in the nucleus and binds to p53, which shuttles it out of the nucleus and represses its ability to induce apoptosis [39]. Tumor protein p53-inducible protein 3 (TP53I3) is induced by the tumor suppressor p53 and is thought to be involved in p53-mediated cell death [40]. Quantitative real-time PCR demonstrated that these p53-transcriptionally regulated molecules, including *CDK1*, *CCNG1*, *CCNE2*, *CCNB2*, Reprimo, *GTSE1*, *PTEN*, *SFN*, *TP53I3* and *DDB2* were upregulated by HATi II in U251 cells, in confirmation of the results of the microarray. Significant dose-dependent increases in the protein levels of p53 and p21 were also observed subsequent to treatment with HATi II. Other proteins downstream of the p53 signaling pathway, such as CDK1, CCNE2, Reprimo, PTEN, SFN and RRM2, were also significantly and dose-dependently upregulated by HATi II.

The p53 pathway has been reported to be involved in a large number of biological processes, including acetylation. P53 was the first non-histone protein shown to be acetylated by HATs, and p53 acetylation is mediated by the p300 and CBP acetyltransferases *in vivo* [41]. Thus, we suggest that inhibition of HATs may lead to deacetylation of p53 and result in transactivation of the p53-regulated genes that regulate apoptosis. However, additional research is required to explore the precise mechanism by which HATi II affects the p53 pathway.

## Conclusions

HATi II inhibits proliferation and induces apoptosis via the caspase-dependent pathway in glioma cell lines, possibly by activating the p53 signaling pathway. This study provides a basis for further investigation of the molecular mechanism by which HATi II induces apoptosis and indicates that HATi II may have potential as a therapeutic option for human glioma.

## Additional files

Additional file 1: Table S1. Primers used in this study. Additional file 2: Figure S1. HATi II induces apoptosis in glioma cell lines. **(A-C)** U87 cells or **(D-F)** HS683 cells were treated with HATi II for 24 or 48 h and apoptosis was determined by Annexin V-FITC/PI dual labeling and flow cytometry **(A, D)**, and the numbers of Annexin V (+) and PI (-)% cells were quantified **(B, E)**; **P* < 0.05 or ***P* < 0.01 compared with DMSO-treated control cells. **(C, F)** Cell cycle analysis of U87 and HS683 cells treated with HATi II for 24 or 48 h; DNA fragmentation was observed after 24 h and increased in a time-dependent manner. These analyses were repeated three times. Additional file 3: Figure S2. HATi II induces apoptosis, DNA fragmentation and activation of caspase-3 in glioma cell lines. **(A, B)** The morphologic changes in U87 **(A)** and HS683 cells **(B)** treated with HATi II were evaluated using Hoechst 33342 staining and fluorescence microscopy. **(C, D)** Apoptosis was assessed by terminal deoxynucleotidyl transferase mediated dUTP nick end-labeling (TUNEL) analysis in U87 **(C)** and HS683 cells **(D)** treated with HATi II. Apoptotic cells (green) were detected by laser scanning confocal microscopy at an excitation of 515-565 nm, while the cell nuclei were stained with DAPI. The two images have been superimposed to show the apoptotic cells (green) and their position. (**E** and **F**) Western blot analysis of the activation of caspase-3, caspase-9 and PARP in U87 **(E)** and HS683 cells **(F)** treated with HATi II for 48 h. Additional file 4: Table S2. Expression profiles of differentially expressed mRNAs in HATi II-treated and DMSO-treated U251 cells. Sheet 1 represents all target values; Sheet 2 is a boxplot view used to compare the distributions of the expression values for the samples in an experiment after normalization; Sheet 3 is a scatterplot, a useful method for assessing the variation between chips; Sheet 4 is the hierarchical clustering, showing distinguishable mRNA expression changes between the samples. Additional file 5: Table S3. Differentially expressed mRNAs in HATi II-treated U251 cells. Sheet 1 represents mRNAs upregulated in HATi II-treated U251 cells and Sheet 2 represents mRNAs downregulated in HATi II-treated U251 cells, compared to DMSO-treated control cells. Additional file 6: Table S4. Expression profiles of differentially expressed lncRNAs in HATi II-treated and DMSO-treated U251 cells. Sheet 1 represents all target values; Sheet 2 is a boxplot view used to compare the distributions of the expression values for the samples in an experiment after normalization; Sheet 3 is a scatterplot, a useful method for assessing the variation between chips; Sheet 4 is the hierarchical clustering, showing distinguishable mRNA expression changes between the samples. Additional file 7: Table S5. Differentially expressed lncRNAs in HATi II-treated U251 cells. Sheet 1 represents lncRNAs upregulated in HATi II-treated U251 cells and Sheet 2 represents lncRNAs downregulated in HATi II-treated U251 cells, compared to DMSO-treated control cells. Additional file 8: Table S6. LncRNAs nearby coding genes. Additional file 9: Table S7. GO analysis of the molecular function of the upregulated mRNAs in HATi II-treated U251 cells. Additional file 10: Table S8. GO analysis of the biological processes related to the upregulated mRNAs in HATi II-treated U251 cells.

## Abbreviations

CBP: CREB-binding protein
PARP: Poly ADP-ribose polymerase
HATs: Histone acetyltransferases
HDACs: Histone deacetylases
HATi II: Histone acetyltransferase inhibitor II
PCAF: P300/CBP-associated factor
DAPI: 4’,6-diamidino-2-phenylindole
p53: tumor suppressor p53
AML: Acute myelocytic leukemia
TUNEL: TdT-mediated dUTP Nick-End Labeling
NSCL: Melanoma and non-small-cell-lung
STK40: Serine/threonine kinase 40
BAG6: BCL2-associated athanogene 6
MMP 2: Matrix metalloprotein 2
MMP 9: Matrix metalloprotein 9
RASSF1: Ras Association (RalGDS/AF-6) Domain Family Member 1
