# Supplementary figures and images for: Histone acetyltransferase inhibitor II induces apoptosis in glioma cell lines via the p53 signaling pathway
Source: J Exp Clin Cancer Res. 2014 Dec 19;33(1):108. doi: 10.1186/s13046-014-0108-3 (PMC4321714; doi:10.1186/s13046-014-0108-3)

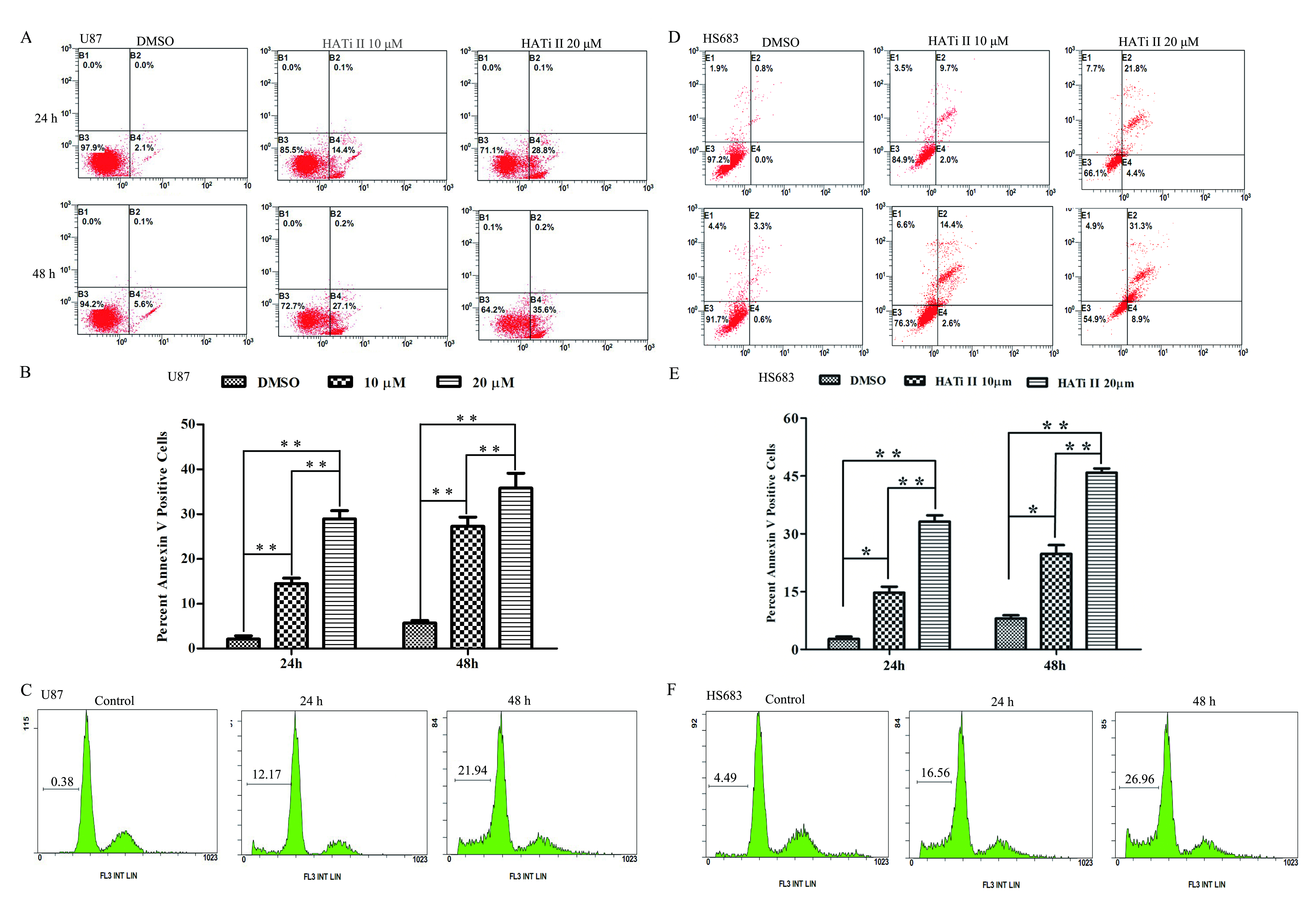

Supplement: Additional file 2: Figure S1. — HATi II induces apoptosis in glioma cell lines. (A-C) U87 cells or (D-F) HS683 cells were treated with HATi II for 24 or 48 h and apoptosis was determined by Annexin V-FITC/PI dual labeling and flow cytometry (A, D), and the numbers of Annexin V (+) and PI (-)% cells were quantified (B, E); *P < 0.05 or **P < 0.01 compared with DMSO-treated control cells. (C, F) Cell cycle analysis of U87 and HS683 cells treated with HATi II for 24 or 48 h; DNA fragmentation was observed after 24 h and increased in a time-dependent manner. These analyses were repeated three times. [file 13046_2014_108_MOESM2_ESM.jpeg]

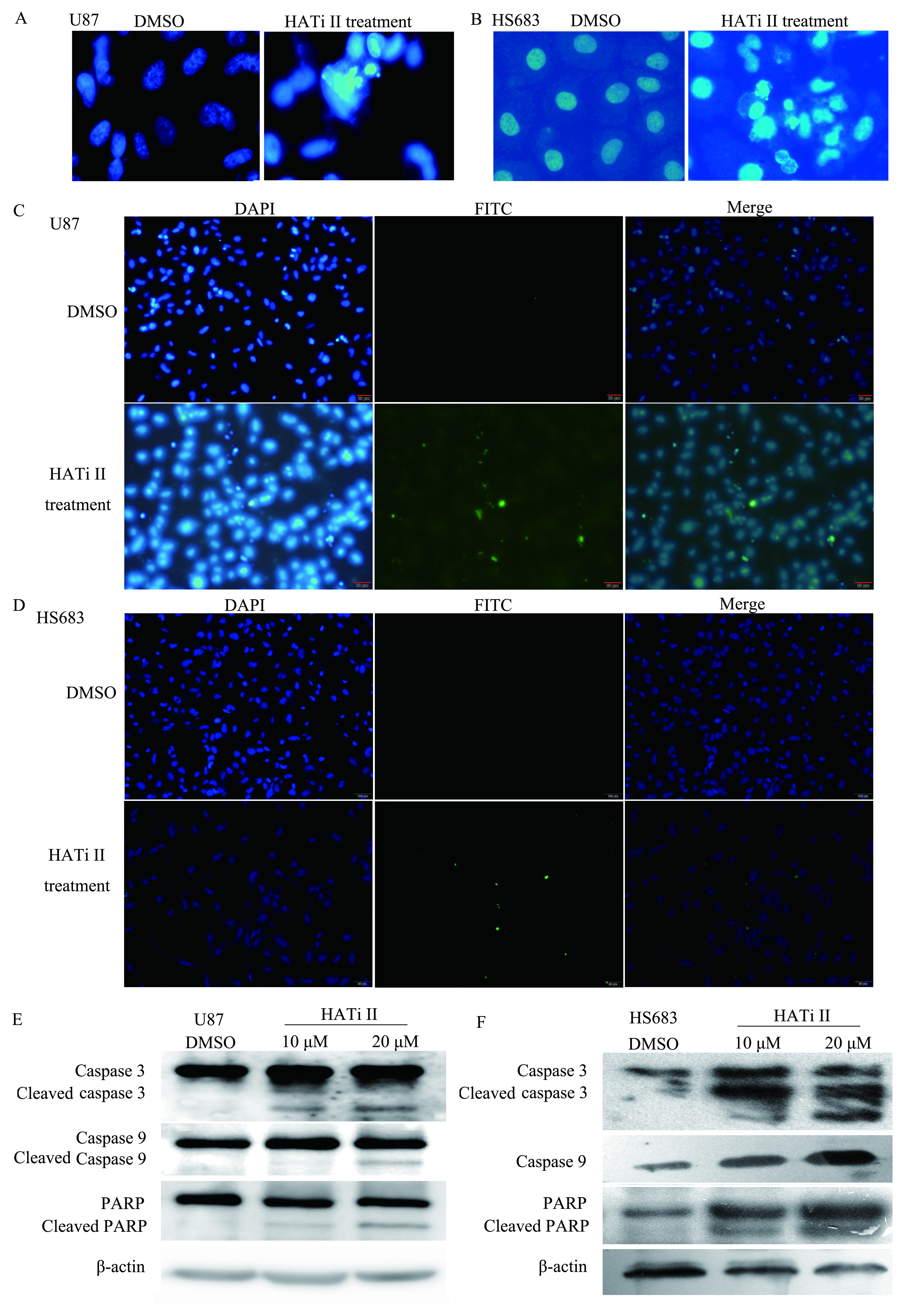

Supplement: Additional file 3: Figure S2. — HATi II induces apoptosis, DNA fragmentation and activation of caspase-3 in glioma cell lines. (A, B) The morphologic changes in U87 (A) and HS683 cells (B) treated with HATi II were evaluated using Hoechst 33342 staining and fluorescence microscopy. (C, D) Apoptosis was assessed by terminal deoxynucleotidyl transferase mediated dUTP nick end-labeling (TUNEL) analysis in U87 (C) and HS683 cells (D) treated with HATi II. Apoptotic cells (green) were detected by laser scanning confocal microscopy at an excitation of 515-565 nm, while the cell nuclei were stained with DAPI. The two images have been superimposed to show the apoptotic cells (green) and their position. (E and F) Western blot analysis of the activation of caspase-3, caspase-9 and PARP in U87 (E) and HS683 cells (F) treated with HATi II for 48 h. [file 13046_2014_108_MOESM3_ESM.jpeg]
